# Convolutional Neural Network Model Based on 2D Fingerprint for Bioactivity Prediction

**DOI:** 10.3390/ijms232113230

**Published:** 2022-10-30

**Authors:** Hamza Hentabli, Billel Bengherbia, Faisal Saeed, Naomie Salim, Ibtehal Nafea, Abdelmoughni Toubal, Maged Nasser

**Affiliations:** 1 Laboratory of Advanced Electronics Systems (LSEA), University of Medea, Medea 26000, Algeria; 2 UTM Big Data Centre, Ibnu Sina Institute for Scientific and Industrial Research, Universiti Teknologi Malaysia, Johor Bahru 81310, Johor, Malaysia; 3 DAAI Research Group, Department of Computing and Data Science, School of Computing and Digital Technology, Birmingham City University, Birmingham B4 7XG, UK; 4 College of Computer Science and Engineering, Taibah University, Medina 41477, Saudi Arabia; 5 School of Computer Sciences, Universiti Sains Malaysia, Gelugor 11800, Penang, Malaysia

**Keywords:** activity prediction model, biological activities, bioactive molecules, convolutional neural network, deep learning

## Abstract

Determining and modeling the possible behaviour and actions of molecules requires investigating the basic structural features and physicochemical properties that determine their behaviour during chemical, physical, biological, and environmental processes. Computational approaches such as machine learning methods are alternatives to predicting the physiochemical properties of molecules based on their structures. However, the limited accuracy and high error rates of such predictions restrict their use. In this paper, a novel technique based on a deep learning convolutional neural network (CNN) for the prediction of chemical compounds’ bioactivity is proposed and developed. The molecules are represented in the new matrix format Mol2mat, a molecular matrix representation adapted from the well-known 2D-fingerprint descriptors. To evaluate the performance of the proposed methods, a series of experiments were conducted using two standard datasets, namely the MDL Drug Data Report (MDDR) and Sutherland, datasets comprising 10 homogeneous and 14 heterogeneous activity classes. After analysing the eight fingerprints, all the probable combinations were investigated using the five best descriptors. The results showed that a combination of three fingerprints, ECFP4, EPFP4, and ECFC4, along with a CNN activity prediction process, achieved the highest performance of 98% AUC when compared to the state-of-the-art ML algorithms NaiveB, LSVM, and RBFN.

## 1. Introduction

Extraction of the structural activity relationship (SAR) [1,2] information from chemical datasets relies on the pairwise structural comparison of all toxicophore features and small molecules, which highlights the degree of the structural relationship between the compounds [3,4,5,6]. The Quantitative Structure–Activity Relationship (QSAR) can correlate the compound’s chemical and structural features with its physicochemical or biological properties. The molecular descriptors are applied for encoding the features, while the QSAR model identifies the mathematical relationship between the descriptors and the biological features or other relevant properties of the known ligands for predicting the unknown ligands. These QSAR studies are able to reduce the failure costs of potential drug molecules, as they easily identify the promising lead molecules and reduce the number of expensive experiments. These are considered important tools in the pharmaceutical industry since they have identified many high-quality leads during the early stages of drug discovery.

A great deal of information is contained in the molecular structure of a compound: For example, it indicates the number of elements or describes its shape and electrostatic field [7,8]. The collection of atoms that constitute a molecule can be symbolically represented in many ways. It is not easy to determine the optimum approach that represents the molecular structure that is suited for all applications [9,10,11].

Generally, molecules are represented using their molecular or structural formulae and line drawings, which indicate the number of atoms for various elements present in the single molecule of a compound, for example, H_2_O indicates the presence of two hydrogens and one oxygen atom in a water molecule. In many cases, the molecular formula alone cannot represent the chemical structure. For instance, in isomers, molecules with a similar molecular formula show a different atomic arrangement. The structural formula depicts the molecular structure and represents the individual bonds between all atoms as lines.

Many chemoinformatics methods are based on numerical descriptors that include a description of the molecular structure and properties. These descriptors are used as input data for various statistical and data mining techniques. The other types of property descriptors are generally used in the diversity analysis, selection of the representative compound subsets, combinatorial library design, and QSAR studies. Thus, the fingerprint X of molecule A is represented using a sequence of numbers:

$$X_{A}=\left\{x_{1},x_{2},x_{3},\;\ldots ,x_{n}\right\}$$

where $x_{i}$ refers to the i-th structural unit in molecule A, i.e., bonds, atoms, or fragments. The value n represents the length or size of all fingerprints, i.e., the number of molecular properties.

**Figure 1:**
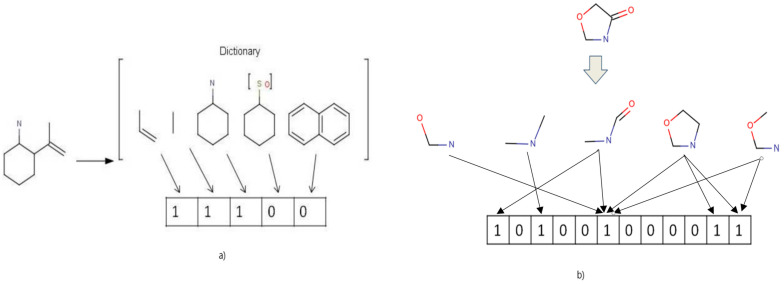
Two examples showing the generation of a molecular fingerprint: (**a**) Dictionary-based fingerprint and (**b**) hashed-based fingerprint.

The 2D fingerprint descriptors are also used to provide a rapid screening step during substructure and similarity searches [1,10]. These 2D fingerprints are categorised based on the methods used, for example, the fragment dictionary and hashed methods illustrated in Figure 1. The fingerprints are generated using a fingerprinting process that converts a chemical structure into a binary form (i.e., a string of 0s and 1s). The binary form depicts the chemical shorthand, which indicates the presence/absence of the structural features in a molecule.

The molecule-based fingerprints are represented by dividing the molecules into fragments of specific substructures and structural features. In this kind of representation, the fingerprint length is based on the number of fragments present in the dictionary, where every bit position in the binary string is assigned to one particular sub-structural feature in the dictionary. Thus, the bits can individually or in combination represent the presence or absence of the features [10,12].

The state-of-the-art 2D fingerprint technique used in the present study was based on QSAR, which can predict and measure all biological activities of the compounds. In this study, eight different 2D fingerprints were investigated for bioactivity prediction, which was generated using the PaDEL descriptor software. Here, the 2D fingerprint descriptors were used with the CNN model for predicting the biological activities and studying the combination and the integration of various fingerprints in the CNN architecture. The next sections describe the background and design of the novel technique. The performance of the proposed technique was evaluated after conducting several experiments based on the structure or bioactivity prediction.

## 2. Results

The proposed code was implemented in public DL software, Keras [13], based on Theano [14]. The experiments were conducted using the Dell Precision T1700 CPU system with 16 GB memory and the professional-grade NVIDIA GeForce GTX 1060 6 GB graphics.

The proposed novel CNN model for predicting the molecular bioactivities was a ligand-based activity prediction or target-fishing technique that could be used for unknown chemical compounds. It was a deep learning system consisting of an adapted molecular matrix representation, “Mol2mat”, which incorporated all the substructural data on the molecules based on their fingerprint features for predicting their activities. This proposed CNN method was then compared to three different ML algorithms described in the WEKA-Workbench, NaiveB, LSVM, and RBFN, using optimal parameters obtained from previous work using the same datasets [15], as previously explained in Section 4.4.

We also determined the computing prediction accuracy of this deep learning system by applying the technique described in Section 4.2, using eight fingerprint representatives. The results derived from these fingerprints were then compared using the Analysis of variance (ANOVA) technique as a significance test and a violin-plot with boxplot charts. The five fingerprint representatives that showed the best CNN configuration were further chosen as the best representatives. This encompassed Stage 1 of the analysis and is described in detail below. In Stage 2, these five representatives were assessed using all probable combinations, such as 2, 3, 4, or 5. The results acquired from Stage 2 were further compared using their violin-plot charts, and the best fingerprint combination was noted. Stage 2 is described in more detail below. In Stage 3, all results were compared for the best combination derived from the previous stages with three known ML algorithms, NaiveB, LSVM, and RBFN. The proposed CNN model in this paper will be henceforth referred to as CNNfp.

### 2.1. Benchmarking

The proposed technique was evaluated by comparing it with three other machine learning methods using WEKA-Workbench [16] methods, including a Naive Bayesian classifier (NaiveB) [17], LibSVM [18], and a Radial basis function network (RBFN) [19]. Finding the best values for the classifier’s parameters is a difficult task. However, the best probable setup for the LSVM classifier was identified by the WEKA-Workbench. In this paper, the linear kernel was used for SVM, and the values of 0.1, 1.0, and 0.001 were used for the Gamma, Cost, and Epsilon parameters, respectively. For the NaiveB classifier, a supervised discretisation technique was used to convert the numeric attributes to the nominal attributes, while the minimal standard deviation limit for the RBFN classifier was 0.01. All the remaining parameters of the classifiers used the default values in the WEKA-Workbench.

### 2.2. Stage 1

**Figure 2:**
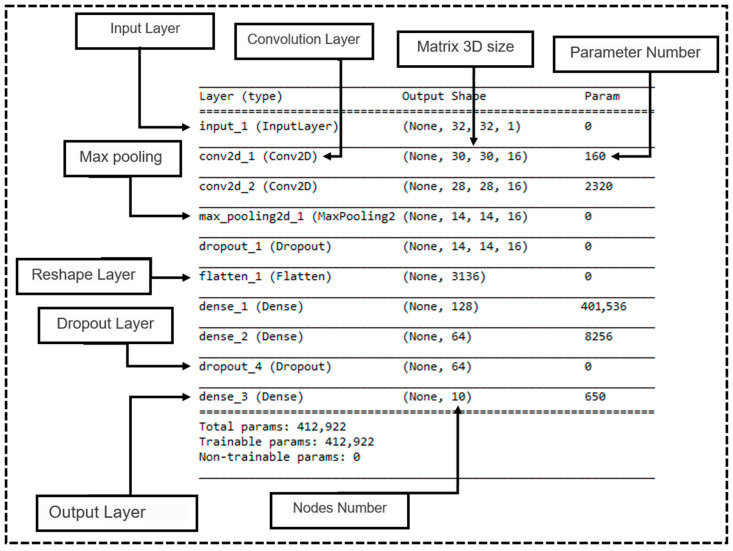
A summary of the proposed CNN configuration that uses the Mol2Mat representation.

In this stage, the prediction accuracies of the 24 activity classes present in an MDDR1, MDDR2, and Sutherland datasets were determined and compared using eight fingerprint representatives. Figure 2 summarises the CNN configuration, which used the Mol2mat molecular representation.

In Stage 1, the eight fingerprints described above were studied based on two parameters. The first parameter included the accuracy response vs. the number of iterations, while the second parameter included the MSE response vs. the number of epochs. These were studied in a 2D graph consisting of the training data results.

**Figure 3:**
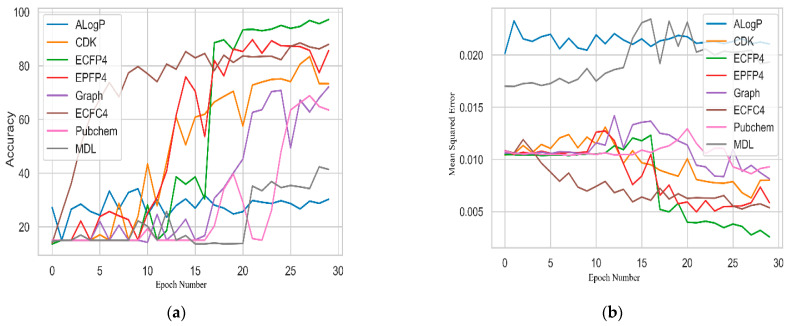
Evaluation of eight fingerprints based on their (**a**) accuracy and (**b**) MSE performance.

Figure 3a presents a graphical result for the number of iterations vs. the accuracy. It also presents eight lines of the different fingerprints. The ECFC4 fingerprint displayed a speed augmentation in their prediction accuracy from the third epoch, whereas the EPFP4 fingerprint showed better accuracy in 17 epochs. However, the AlogP and the MDL fingerprints displayed the lowest prediction accuracy values. The mean squared error or loss value showed similar results to the accuracy performance, as shown in Figure 3b. The novel CNN model could accurately predict biological activities with an average MSE value of 0.0054 for ECFC4 and 0.002 for the ECFP4 fingerprints.

**Figure 4:**
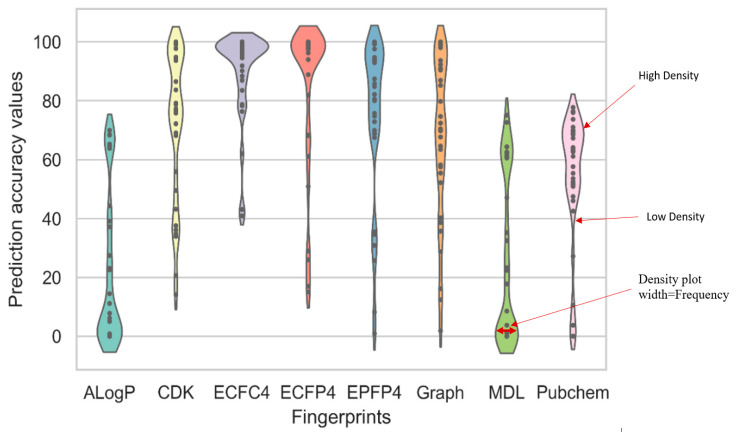
Prediction accuracy values of the CNN model for the eight fingerprint representatives using the violin-plot charts.

Figure 4 shows the comparison of the prediction accuracy values for Stage 1 experiments that were conducted using the CNN model for eight fingerprint representatives using the violin-plot charts. The construction of violin-plot charts is shown on the right-hand side of this figure.

The violin-plot charts are able to remove the conventional boxplot elements and plot each activity class as a single point. Figure 2 indicates that the eight fingerprint representatives showed a clear difference in their average prediction accuracy values. The ECFC4 showed the best average accuracy of 90.17. The graph fingerprint came next with a value of 74.84, closely followed by the CDKFp and ECFP4 fingerprints, which showed similar average accuracy values of 72.28 and 71.97, respectively. The worst average accuracy values were displayed by PubChem (53.88), MDL 26.25, and AlogP, with an accuracy value of only 22.45. Using these results, and based on the ANOVA significant test results, a small *p*-value of 0.04 was noted, which highlighted the difference between all the fingerprints.

Furthermore, the AlogP, MDL, and PubChem fingerprints were regarded as the worst contenders as they showed a higher variance between all the biological activity classes. Thus, CDK, ECFP4, ECFC4, EPFP4, and graph were some of the best fingerprints and could be forwarded to Stage 2 to improve all the results based on the probable combination cases of two, three, four, or five of the best fingerprints. The combinations were based on the fusion of the extracted feature levels.

In this stage, we used better techniques to combine the various sources of knowledge available in the area of deep learning [20,21,22]. Firstly, we proposed a feature extraction step to present each selected molecular fingerprint. This combination significantly improved the models, since they could benefit from every molecular fingerprint and combine all the extracted features from various sources after a flattened layer, which followed the max-pooling layer. This helped them convert the 2D matrix data into the vector. As a result, they could process the output data using the fully connected layers, known as the dense layers. This section described the CNN architecture utilised in this study and how many CNN architectures can be combined into a single model. The next section will describe the performance evaluation.

### 2.3. Stage 2

In this stage, the prediction accuracies for the different combination cases of the five fingerprint representatives were determined. Table 1 presents 26 possible combinations for these five fingerprints, including combinations of two, three, four, and five combinations of the CDK, ECFP4, ECFC4, EPFP4, and graph fingerprints. Henceforth, each combination case will be based on its name (A–Z), and each row will represent one combination case. Case A consists of two combinations, while Case Z consists of five fingerprint combinations.

**Figure 5:**
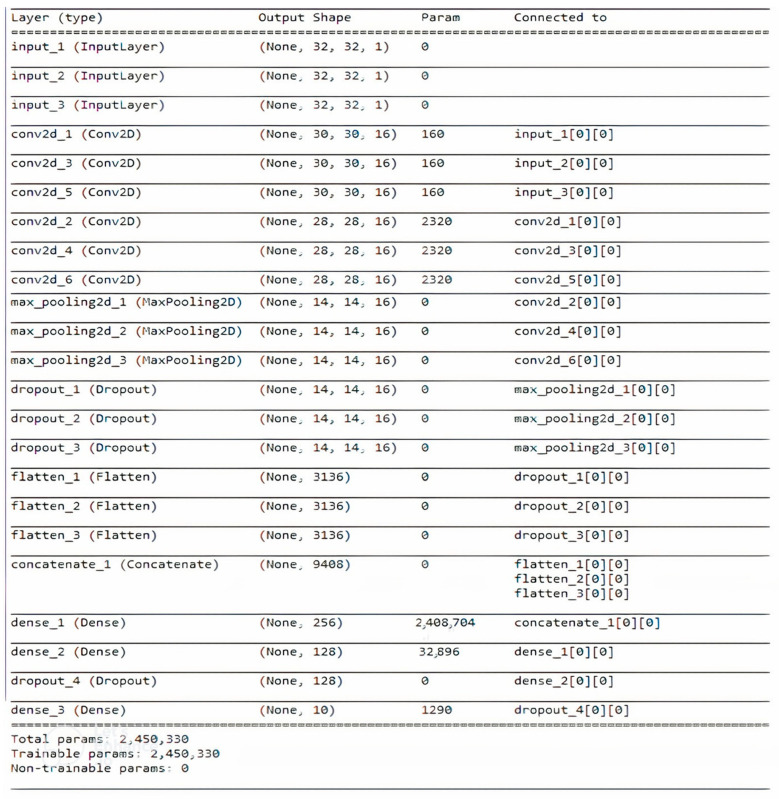
A summary of the CNN configuration for a combination case named “K” using a Mol2mat representation.

**Figure 6:**
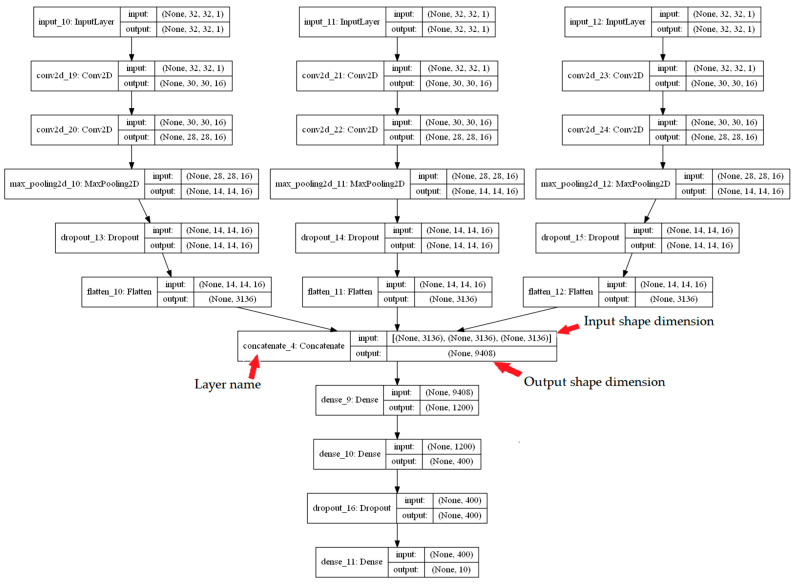
A CNN Model configuration for a combination case named “K” using the Mol2mat representation.

The 26 combinations of the five fingerprints were investigated, as shown in Table 1. Figure 5 and Figure 6 summarise the CNN configuration for the combination case between the CDK, ECFP4, and EPFP4 fingerprints, referred to as “K”, as an example using the Mol2mat molecular representation. As seen in both figures, the model has three branches, with a matrix (32 × 32) as the input and two Conv. layers and max-pooling for each branch concatenate layer to merge all extracted features into one array. Finally, there are two hidden layers with 256 and 128 neurons and an output layer with 10 outputs. Rectified linear activation functions are used in each hidden layer, and a SoftMax activation function is used in the output layer.

**Figure 7:**
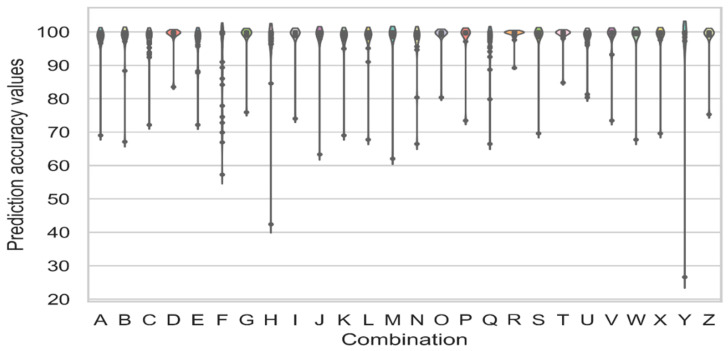
Prediction accuracy values for the CNN model were applied to the 26 combination cases of the five best fingerprints with the help of violin-plot charts.

Figure 7 compares the prediction accuracy values for the Stage 2 experiments for all 26 combination cases with the help of violin-plot charts.

**Figure 8:**
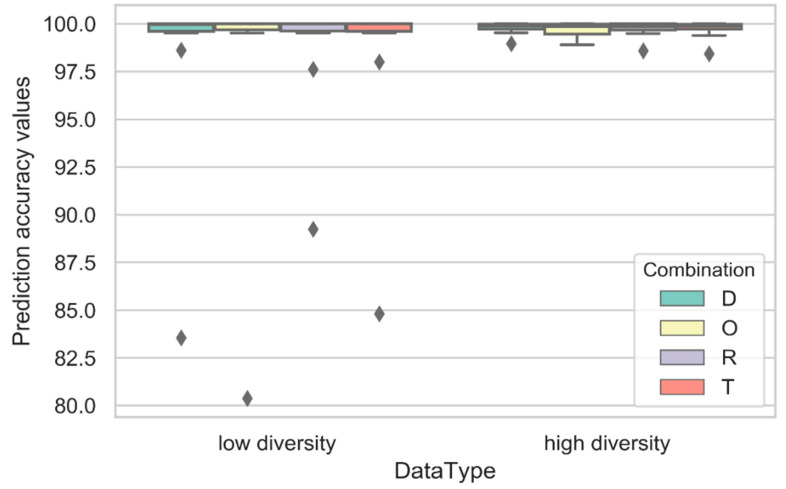
A comparison of the prediction accuracies for the D, O, R, and T combination cases, plotted using the boxplot charts.

The results in Figure 7 show a *p*-value of 0.031 based on the ANOVA significance test results, indicating that the difference between all the combination cases is significant. The violin-plot charts plotted each activity class as the point. It was seen that the D, O, R, and T combination cases displayed the highest prediction accuracy, >80%, and a low variance amongst all the activity classes. The combination cases were plotted in different boxplot charts to determine the distribution of the activity classes based on the low- and high-diversity values noted for each activity class. Figure 8 compares the prediction accuracies for all experiments in Stage 2 for the D, O, R, and T combination cases, which were plotted using the Boxplot charts.

Based on the violin-plot charts presented in Figure 7 and the Boxplot chart shown in Figure 8, a *p*-value of 0.048 was calculated based on the ANOVA significance test results. This indicated the significance of the difference between all the models. The R combination displayed the best average prediction accuracy of 99.17, indicating that a combination of the three fingerprints, ECFP4, EPFP4, and ECFC4, showed good performance compared to the other combinations.

The R combination also showed a lower variance of 5.52 compared to the other cases. Furthermore, this combination showed higher stability even when placed in a high- or low-diversity class. Meanwhile, the D, O, and T combinations displayed a mean prediction accuracy of 97.45, 97.03, and 97.72, respectively. They also displayed higher variance than the R combination. These combination cases showed a variance prediction accuracy of 12.62, 17.97, and 10.81, respectively, indicating that R was the best fingerprint combination seen in Stage 2.

### 2.4. Stage 3

In Stage 3, the authors compared the results for the best combination of ECFP4, EPFP4, and ECFC4, as established in Stage 2, with those obtained from the standard ML algorithms existing in a WEKA-Workbench: NaiveB, LSVM, and RBFN.

Table 2, Table 3 and Table 4 show the sensitivity, specificity, and AUC values for all the datasets used here. A visual inspection of all tables could be used to compare the performance of the prediction accuracies of all four algorithms. However, the authors applied a quantitative boxplot chart to compare these algorithms. This process quantifies the agreement level between all the multiple sets and ranks the different objects.

Boxplot charts were used to assess the performance of a set of fingerprints, ECFP4, EPFP4, and ECFC4, using three algorithms (RBFN, NaiveB, and LSVM).

**Figure 9:**
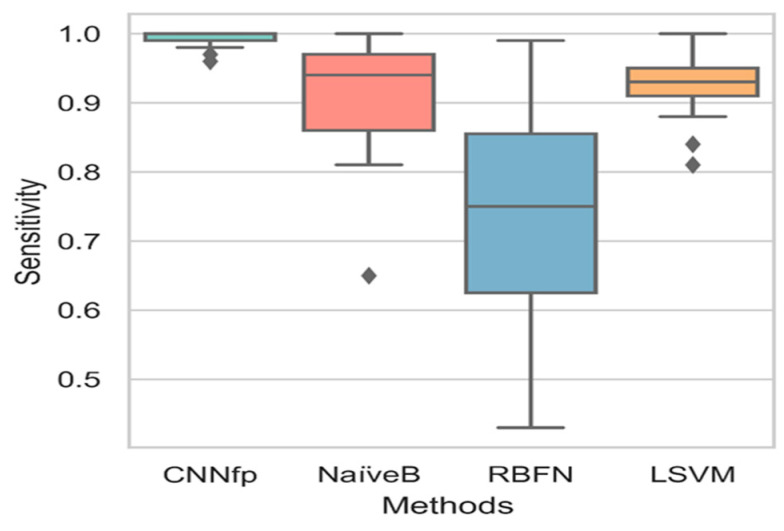
Boxplot chart results based on comparing the sensitivity values of different algorithms: CNNfp, NaiveB, RBFN, and LSVM.

Here, MDDR1, MDDR2, and the Sutherland datasets, with their activity classes described in Table 5, Table 6 and Table 7, were regarded as judges. In contrast, parameters such as sensitivity, specificity, and AUC, measured using different prediction algorithms, were regarded as objects. The outputs of this test included *p*-value, median, and variance. Figure 9 shows the results of the boxplot chart, where the sensitivity values of the six algorithms were compared. The results show a *p*-value of 0.008 based on the ANOVA significance test results, which revealed a significant difference between all algorithms. The CNNfp algorithm showed a high sensitivity of 0.985, while the NaiveB and LSVM ML algorithms showed a high variance of 0.15 and 0.23, respectively, compared to the CNNfp. Diversity in all sensitivity values was especially seen in the algorithms that displayed a variance of 10^−4^. Furthermore, these models showed a mean sensitivity of 0.90 and 0.74, respectively.

**Figure 10:**
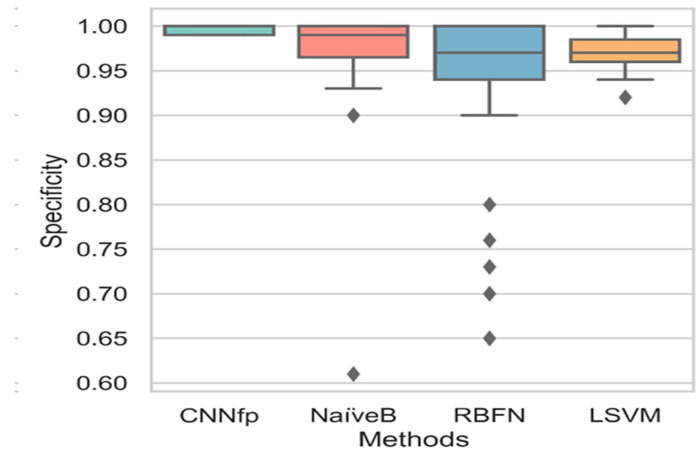
Boxplot chart results based on comparing the specificity values of different algorithms: CNNfp, NaiveB, RBFN, and LSVM.

Figure 10 shows the boxplot chart results after comparing the specificity values of the CNNfp, NaiveB, RBFN, and LSVM algorithms. The NaiveB and RBFN ML algorithms showed a higher variance of 0.01 and 0.04, respectively, compared to the CNNfp. This diversity in all specificity values was especially seen in the algorithms that displayed a variance of 2.5 × 10^−5^. Furthermore, the CNNfp algorithm showed a high specificity value of 1.0, whereas the NaiveB and the RBFN algorithms displayed average specificity values of 0.99 and 0.98, respectively. The results showed a small *p*-value of 3.5 × 10^−5^, highlighting a significant difference between all algorithms.

**Figure 11:**
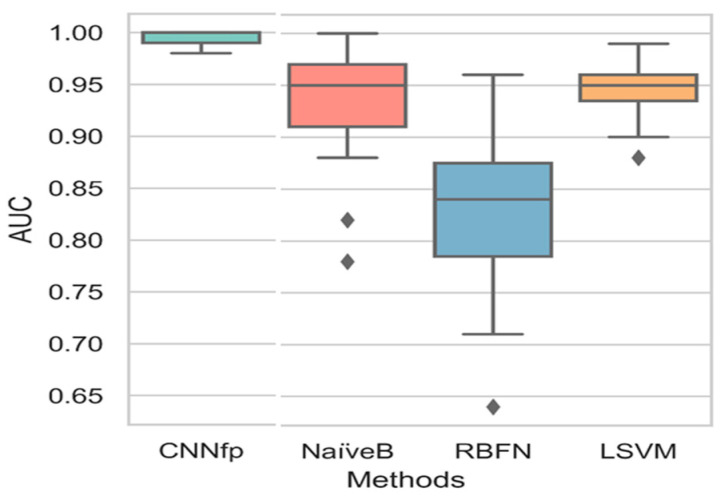
Boxplot chart results based on the comparison of the AUC values of different algorithms: CNNfp, NaiveB, RBFN, and LSVM.

Figure 11 describes the Boxplot chart results after comparing the AUC values of the CNNfp, NaiveB, RBFN, and LSVM algorithms. The LSVM, NaiveB, and RBFN ML algorithms showed a higher variance of 0.125, 0.083, and 0.033, respectively, compared to CNNfp. This diversity in all AUC values was especially seen in the algorithms that displayed a variance of 4.13 × 10^−5^. A combination of the Mol2mat with the CNNfp algorithm showed an AUC value of 0.99, whereas the LSVM, NaiveB, and RBFN algorithms displayed higher average AUC values of 0.96, 0.99, and 0.85, respectively. The results showed a *p*-value of 4.2 × 10^−3^, highlighting a significant difference between all algorithms.

The boxplot chart results (Figure 9, Figure 10 and Figure 11) showed that the use of CNNfp was very efficient and convenient and presented less severe outliers in comparison to the NaiveB, RBFN, and LSVM algorithms, thereby indicating the effectiveness of this prediction approach. The results presented in Table 2, Table 3 and Table 4 for all three datasets show that the combination of ECFP4, EPFP4, and ECFC4 fingerprints with a CNN activity prediction method resulted in the lowest variance for the sensitivity, specificity, and AUC values for all activity classes compared to the traditional NaiveB, RBFN, and LSVM algorithms. These results suggest that a deep learning technique could be a promising, novel, and effective method of predicting the activities of a range of chemical compounds.

## 3. Discussion

### 3.1. Similarity Searching

Comparing unknown molecules to known chemical compounds allows us to predict the activities of targets that are unknown compounds. Thus, the target compounds will exhibit the activities of similar compounds. Several successful target prediction techniques have been proposed in the literature [11,23,24]. For example, the authors in [25] implemented a method for activity prediction using the Multi-level Neighbourhoods of Atoms (MNA) structural descriptor. This descriptor is generated based on the connection table and the table of atoms that represent each compound. A specific integer number is given to each descriptor according to its dictionary. The Tanimoto coefficient was effectively used to calculate the molecular similarity. The target compound activities were then predicted based on the activities of the most similar known compound.

A number of machine learning techniques have been used for activity prediction (target), including Binary Kernel Discrimination (BKD), Naive Bayesian Classifier (NBC), Artificial Neural Networks (ANN), and Support Vector Machines (SVM). The authors of [26] predicted five different ion channel targets using BKD and two different types of activity data. They found that the effectiveness of the model increased using highly similar activity classes. However, if this similarity was too low, the models would not work. As it is simple to build a network to include many sources of significant information about molecular structure, the authors of [27] used data fusion to aggregate the results of BIN searches using multiple reference structures. The authors in [28] presented a new classifier of Kinase Inhibitors using the NBC model. One advantage of this method that was noted is finding compounds that are structurally unrelated to known actives or novel targets for which there are inadequate data to develop a specific kinase model. In [29], the authors summarised how networks could conduct the equivalent of discriminant and regression analyses and underlined how initial overtraining and overfitting could lead to poor prediction performance. According to their predictions, the next revolution in QSAR will focus on developing better descriptors for connecting chemical structure to biological activity. The authors of [30] created a set of SVM classifiers that collectively account for 100 different forms of drug molecule action.

In their study, the multilabel-predicted chemical activity profiling was successfully accomplished by SVM classifiers, and they suggest that the proposed approach can forecast the biological activities of unidentified chemicals or signal negative consequences of drug candidates. In [11,31], the Bayesian belief network classifier was applied to predict the compound’s target activities. The authors applied a novel technique to extend previous work, based on a convolutional neural network that uses the 2D fingerprint representation to predict the possibly bioactive molecules. The proposed CNN model for activity prediction also included the substructural information of the molecule.

### 3.2. Convolutional Neural Network for Biological Activity Prediction

In [32], the authors used Merck’s drug discovery datasets and showed that Deep Neural networks (DNN) could obtain better prospective predictions than the existing machine learning methods. In addition, The Multi-Task Deep Neural Network (MT-DNN) model [33] demonstrated good performance by training the neural network with a number of output neurons, where the input molecule’s activity is predicted by every neuron using different assays. In addition, [34,35,36] demonstrated how MT-DNN may be scaled to incorporate big databases such as PubChem Bioassays [37] and ChEMBL [38].

However, several issues and limitations still exist with the current methods. For instance, these methods work with targets that already have more available data and, thus, they cannot predict novel targets. Additionally, the current DL approaches rely on fingerprints, such as ECFP [39], which limit feature discovery to the composition of the particular chemical structures identified by the fingerprinting process [10,34,40]. This reduces their ability to discover arbitrary features. Moreover, the existing DL methods are blind to the target, as they are not able to elucidate the potential molecular interactions.

Another commonly used method is applying the similarity principle [41], which claims that substances with similar structures have similar biological characteristics. However, the authors in [42] discovered that it frequently fails because minor structural modifications can diminish the ligand’s pharmacological activities that describe the molecular similarity within the substructures.

In order to address these issues and limitations, a novel Convolutional Neural Network (CNN)-based model using a 2D Fingerprint was proposed in this study for bioactivity prediction. This technique can be used for several applications such as bioactivity prediction, molecular searching, molecular classification, and virtual screening. The next section provides a description of how the suggested strategy was developed.

## 4. Materials and Methods

This section explains how this model is used for identifying and predicting the bioactivities of chemical compounds. First, we describe how various experimental benchmarks can be built and then utilised for system testing. Next, we discuss the systems for input representation and data encoding and deep convolutional network architecture.

### 4.1. Data Sets

The proposed prediction model was experimentally evaluated using multiple datasets. This study used three datasets (Table 5, Table 6 and Table 7), which were described earlier in [43,44] and used in several studies for validating the ligand-based virtual screening methods [7,11,24,31,45,46].

**Figure 12:**
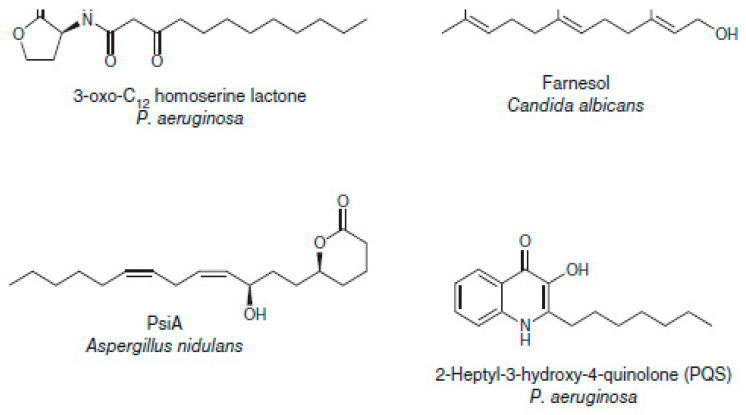
Examples of low-diversity molecules in the MDDR dataset.

**Figure 13:**
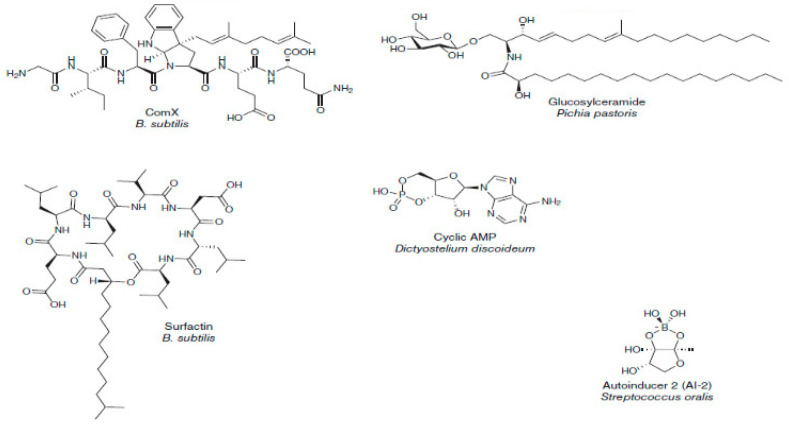
Examples of high-diversity molecules in the MDDR dataset.

The datasets used are disparate, including a structurally homogeneous dataset, as shown in Figure 12, and a structurally diverse dataset, as shown in Figure 13 [3].

**Figure 14:**
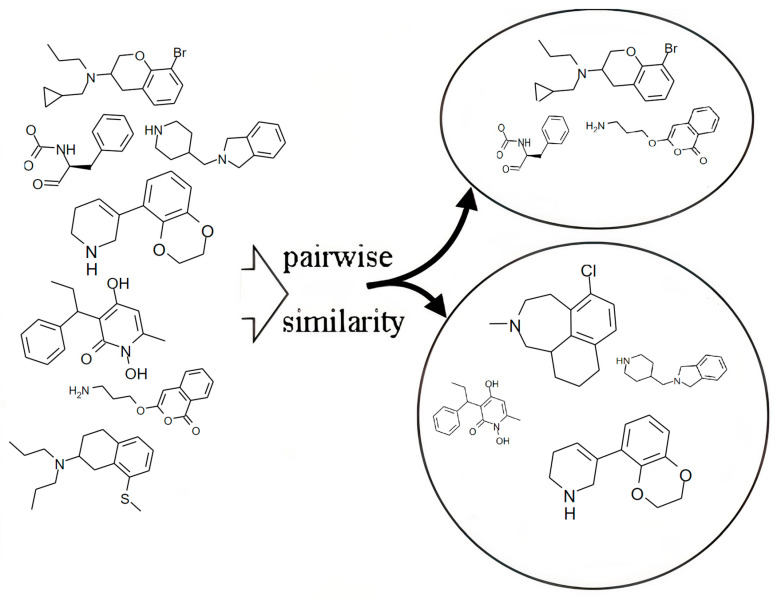
The average pairwise similarity (MPS) across each set of active molecules.

The original version of the MDDR database includes 707 distinct activity classes. The mean pair-wise similarity (MPS) was then computed for each activity class. The mean pair-wise similarity (MPS) of each set of active molecules was used to estimate the diversity. The mean pairwise similarity (MPS) for 102,000 compounds selected randomly from MDDR was 0.200. Figure 14 presents how the MPS can divide the dataset into high- and low-diversity active classes, so that the cut-off point between the high- and low-diversity groups is equal to 0.200. This method is briefly explained and demonstrated in [3].

These datasets, MDDR1 and MDDR2, comprise 10 homogeneous and heterogeneous activity classes; the Sutherland dataset comprises four activity classes each. Table 5, Table 6 and Table 7 list the activity classes, molecules in each class, and diversity between classes. These tables were created using ECFP4 to estimate the mean pairwise Tanimoto similarity across all of the chemical pairs within each class (extended connectivity).

**Figure 15:**
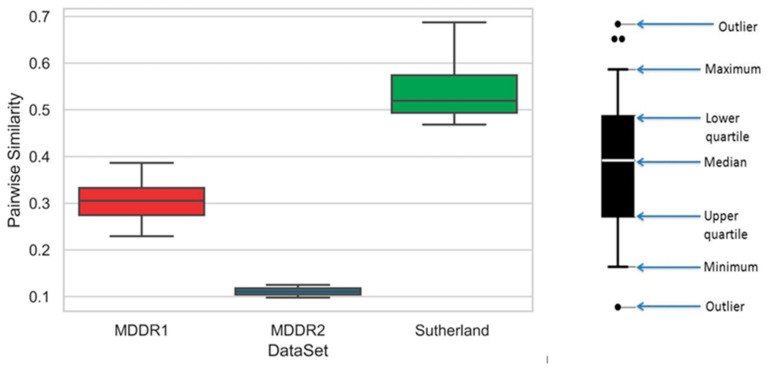
Comparison of MPS values of the three databases using boxplot.

As noted above, the MPS values identify the diversity of activity classes that are used to evaluate the similarity search methods and biological activity prediction. Thus, the MPS values were used to compare the three used databases, as shown in Figure 15.

Box plots are the chart type that is used to visually present the distribution of all numerical data based on their average values and quartiles (or percentiles). Generally, box plots are applied in descriptive statistics since they help in overviewing the set of distributed data along with its range. The right-hand side of Figure 15 depicts the creation of a box, while the median MPS value is represented by the medium segment in the box. The first and third quartiles’ MPS values are shown in the lower quartile and the upper quartile, while an empty circle represents the outlier.

### 4.2. Input Representation

One of the major issues affecting chemoinformatics and QSAR applications is the need for good input features. The general graph-based storage format for chemical compounds’ numerical properties can be calculated using a variety of techniques. Fingerprints are a specific type of complex descriptor that detects the feature distribution from the bit string representations [3]. However, a feature extraction step was necessary to analyse the data in the machine learning technique. The performance of all learning algorithms is enhanced by this stage, which aids in expressing the interpretable data in the machines. Even the best algorithms may perform poorly if the wrong features are used, while simple techniques also perform well if suitable features are applied. Feature extraction techniques can be unsupervised or manually conducted. Here, the authors have presented a new molecular representation, Mol2mat (molecule to matrix), used to reshape each fingerprint molecule representation into a 2D array malleable for use in deep learning architecture.

In this study, the authors investigated eight different 2D fingerprints that were generated using Scitegics Pipeline Pilot software [47]. These included the 120-bit ALOGP, 1024-bit CDK (CDKFP), 1024-bit Path Fingerprints (EPFP4), 1024-bit ECFP4, 1024-bit ECFC4, 1024-bit Graph-Only Fingerprints (GOFP), 881-bit PubChem Fingerprints (PCFP), and the 166-bit Molecular Design Limited (MDL) fingerprints. Table 8 describes the storage of the fingerprint representatives for every molecule in a 2D array, with the help of the row-major order, and also describes every matrix representation Mol2mat size for each fingerprint.

**Figure 16:**
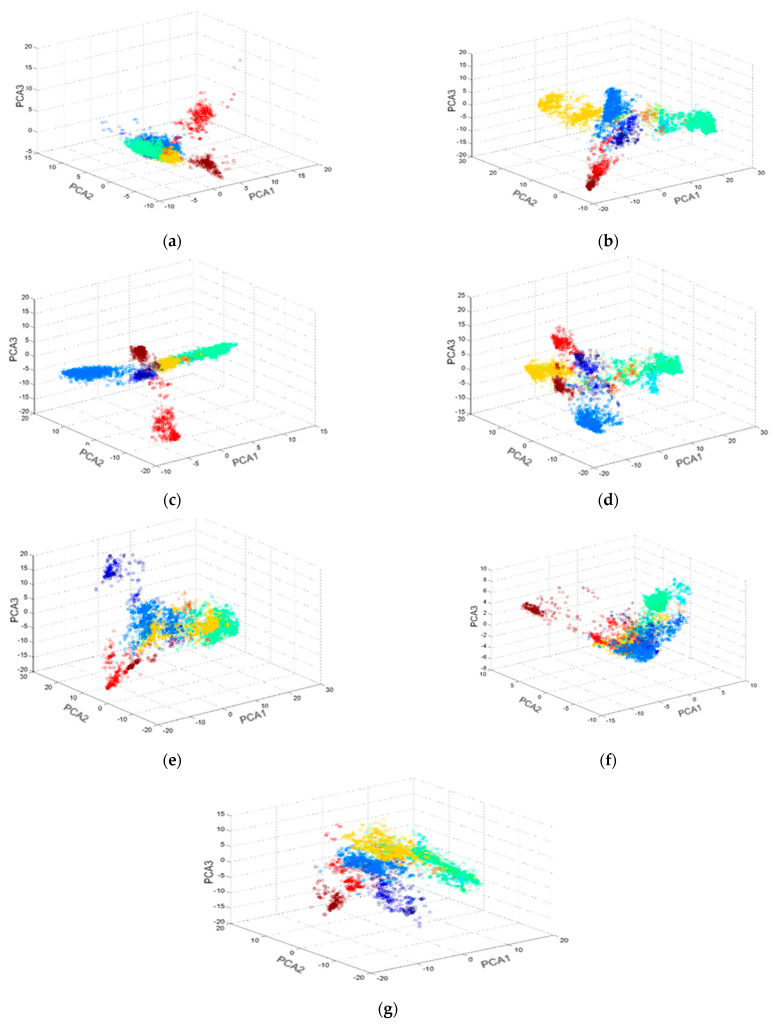
3D−scatter plots based on seven fingerprints and representations of descriptors: (**a**) ALogP, (**b**) CDKFp, (**c**) ECFP4, (**d**) EPFP4, (**e**) GraphOnly, (**f**) MDL, and (**g**) PubchemFp of 5083 different molecules that were selected from the 10 biological activity classes of the MDDR dataset.

To show the difference between different 2D fingerprint representations used in this paper, the authors plotted the scatter graphs in Figure 16 using 5083 molecules (from the MDDR dataset) that are grouped into ten activity classes. These scatter plots were used to establish the relationships between the various compounds belonging to the same class. The molecules were represented by different individual 2D fingerprints and descriptors. In addition, to represent their features, the representation was reduced to a 3D structure using the Principal Component Analysis (PCA) method.

As shown in Figure 16, the ECFP4 2D fingerprint representation can be easily observed and was not overlapping. In addition, the molecules’ biological activities can be segregated. This shows that the suggested 2D fingerprint representation may be successfully used for predicting the biological activity of various chemical substances.

After the generation of the eight fingerprints, the molecular fingerprints were stored in a 2D array using the row-major order, as shown in Algorithm 1.

Algorithm 1 is A summary of the storage of the fingerprints in a 2D array to yield the Mol2mat presentation.**Algorithm 1**: Storing fingerprint in a 2D array
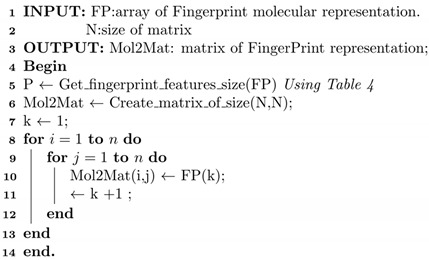


**Figure 17:**
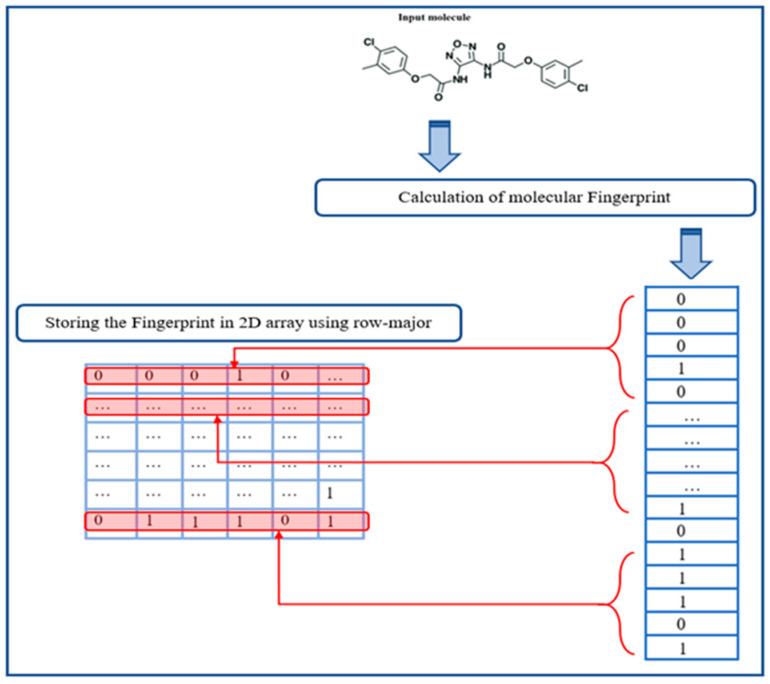
A summary of the newly proposed Mol2mat presentation process.

Algorithm 1 summarises the storage of the fingerprint in a 2D array using the row-major order in pseudo-code form. The algorithm’s output was a 2D array of Mol2mat representations of the input molecule. Figure 17 summarises the design of the Mol2mat presentation process.

After evaluating each fingerprint, the authors assessed all the probable combinations based on the five best descriptors. The combinations were based on the fusion of the extracted feature levels. The combination of multi-CNN can be performed as illustrated in [48,49]. Initially, the combination cases for 2, 3, 4, and 5 were generated by selecting two fingerprints, then three, followed by four, and finally, all five. Thereafter, the best combination was chosen.

### 4.3. Convolutional Neural Network

**Figure 18:**
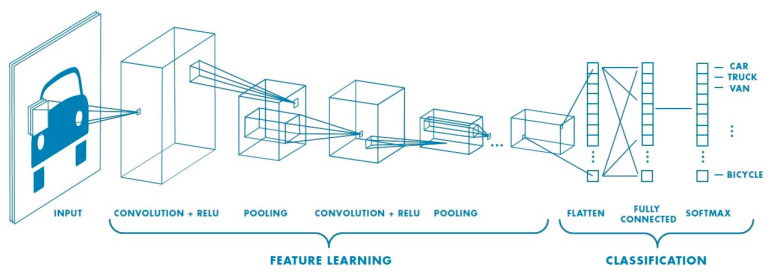
The general CNN configuration.

The default architecture was seen to be a convolutional architecture with fully connected layers. The authors used the Krizhevsky principles [50] for designing the CNN model configuration that was used for viewing the source code [51]. This configuration followed the earlier generic design [50]. Figure 18 presents the general CNN configuration, where the image was passed through the stack of convolutional (conv.) layers. The convolution step employed a max-pooling layer. It was observed that this combination improved the accuracy model and enhanced the CNN configuration.

The flattened layer came after the max-pooling layer. This transformed the 2D matrix data into a single vector, assisting in processing the output that had dense layers, i.e., fully connected layers. The final layer was made of the classification Softmax layer [52,53].

Although CNN displayed good results for the feature learning and the prediction tasks, recent studies have shown a better performance by fusing different CNNs [20,21,54,55]. These combinations can be implemented using feature concatenation or by computing the average or output prediction scores derived from various CNNs.

**Figure 19:**
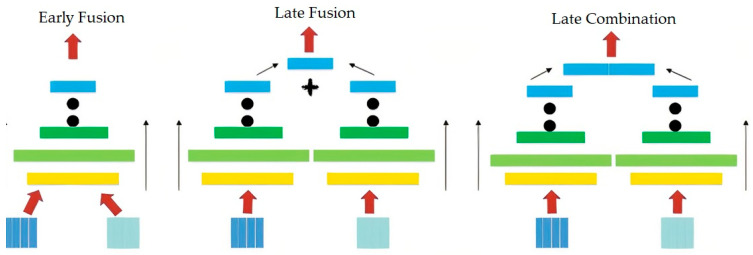
Different approaches used for fusing the information present in the CNN layers.

Some studies [48,49] described the combination of 3 CNN models, as shown in Figure 19. These models were based on the fusion of the information level. Fusion could be performed early in the network after modifying the 1st-layer convolution filters for an extension of time, or it could be performed later, after placing 2 different single-frame networks and then fusing their outputs after the processing. The yellow, green, red, and blue boxes depict the fully connected, normalisation, convolution, and pooling layers, respectively. In a Slow Fusion model, the highlighted columns share the parameters.

In this stage, we used better techniques to combine the various sources of knowledge available in the area of deep learning [20,21,22]. Firstly, we proposed a feature extraction step for presenting every selected molecular fingerprint. This combination significantly improved the models, since they could benefit from every molecular fingerprint and then combine all the extracted features from various sources after a flattened layer, which followed the max-pooling layer. This helped them convert the 2D matrix data into the vector. As a result, they could process the output data using the fully connected layers, called the dense layers. In this section, we described the CNN architecture used in this research and how we can combine multi CNNs in one model. In the next section, we will describe the performance evaluation.

### 4.4. Network Architecture

As mentioned above, eight fingerprint representatives were generated using the Scitegics Pipeline Pilot software [47]. They were further stored in the 2D array with a row-major order for deriving a novel matrix representation Mol2mat, which used the above-mentioned algorithm.

As previously stated, a few fingerprints complemented one another, and their combination yielded good results. This indicated that different fingerprints generated differing results with regard to biological activity prediction or similarity searches. This further indicated that the different QSAR models could be developed based on different fingerprints with similar accuracy. Currently, researchers tend to combine and merge all fingerprints and descriptor sets, which comprise various types of fingerprints [3]. After evaluating each fingerprint, the authors assessed all the probable combinations based on the five best descriptors. The combinations were based on the fusion of the extracted feature levels.

In the present study, we used better techniques for combining the various sources of knowledge available in the area of deep learning [20,21,22]. Firstly, we proposed a feature extraction step for presenting every best molecular fingerprint in which all molecules were passed through 2 conv. layers, using a (3 × 3) feature map size for convolution and one max-pooling layer. This combination significantly improved the models since they could benefit from every molecular fingerprint and combine all the extracted features from various sources after a flattened layer. As a result, they could process the output data using the fully connected layers. The first two fully connected layers were built using a different number of nodes in every combination. Table 9 presents these node numbers in detail in every combination. The combination cases for 2, 3, 4, and 5 were generated by selecting two fingerprints, then three, followed by four, and finally, all five. The best combination was then chosen.

**Figure 20:**
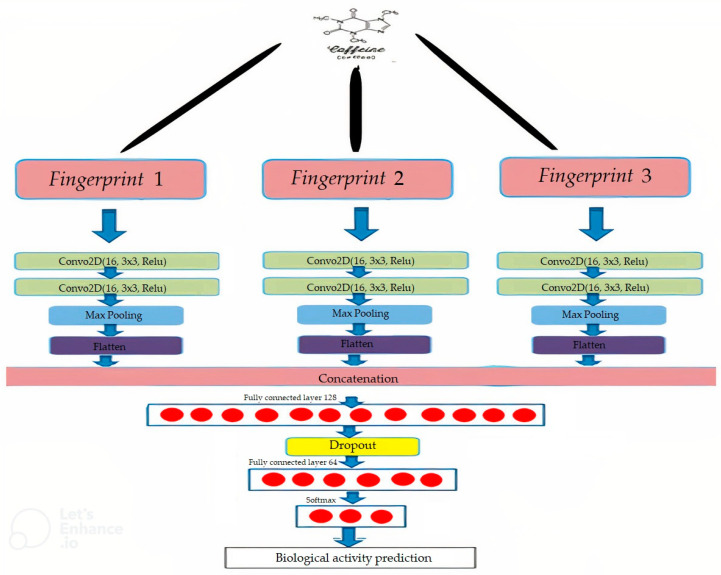
The configuration of the combined CNN that was used for 3 fingerprints.

The final layer included the Softmax layer [50,52,53]. Figure 20 describes the configuration of the combined CNN, which was used to assess 3 fingerprints.

The target was as follows: to predict if the specific chemical compound, *i*, showed activity for target, *t*. These data could be encoded in the binary form, y_it_, where y_it_ = 1 for the active compound and y_it_ = 0 for the inactive compound. This also included the prediction of the compound’s behaviour from targets, simultaneously. In the training stage, a general back-propagation algorithm was used to determine the CNN and decrease the cross-entropy of all targets and the activation of the output layer.

## 5. Conclusions

This study has investigated the use of molecular fingerprinting in the Convolution Neural Network model to predict the activities of ligand-based targets. The results indicate that the combination of the ECFP4, EPFP4, and ECFC4 fingerprints with a CNN activity prediction method produced the lowest variance for the sensitivity, specificity, and AUC values for all the activity classes, when compared to the three traditional ML algorithms of NaiveB, LSVM, and RBFN, available in the WEKA-Workbench. The paper described a novel Mol2mat process, which showed low overlap and was able to segregate all the biological activities of the chemical compounds. A combination of three fingerprints with CNN was used on some popular datasets, and the performance of this combination was compared to that of three traditional ML algorithms. The proposed algorithm achieved good prediction rates (where the low- and high-diversity datasets displayed a 98% AUC value). The results also showed that combining the ECFP4, EPFP4, and ECFC4 fingerprints with CNN improved the performance of both the heterogeneous and homogeneous datasets. In this study, the authors have shown that this combination of fingerprints with the CNN technique is a convenient and stable prediction process, which could be used for determining the activities of unknown chemical compounds. However, this field needs to be investigated further, and better accuracy prediction processes must be developed for high-diversity activity compounds.

## Figures and Tables

**Table 1:** Probable combination cases for the five best fingerprints.

Labels	Combination	CDK	ECFP4	EPFP4	Graph	ECFC4
A	2	√	√			
B	2	√		√		
C	2	√			√	
D	2	√				√
E	2		√	√		
F	2		√		√	
G	2		√			√
H	2			√	√	
I	2			√		√
J	2				√	√
K	3	√	√	√		
L	3	√	√		√	
M	3	√	√			√
N	3	√		√	√	
O	3	√		√		√
P	3	√			√	√
Q	3		√	√	√	
R	3		√	√		√
S	3		√		√	√
T	3			√	√	√
U	4	√	√	√	√	
V	4	√	√	√		√
W	4	√	√		√	√
X	4	√		√	√	√
Y	4		√	√	√	√
Z	5	√	√	√	√	√

The colors are used to differentiate between each level. Combination of 2 blue; Combination of 3 orange; Combination of 4 yellow; Combination of 2 green.

**Table 2:** Sensitivity, specificity, and AUC values for all the prediction models using an MDDR1 dataset.

Activity Index	CNNfp			NaïveB			RBFN			LSVM		
	Sens	Spec	AUC	Sens	Spec	AUC	Sens	Spec	AUC	Sens	Spec	AUC
7707	1.00	1.00	1.00	0.99	1.00	0.99	0.63	1.00	0.82	0.93	0.95	0.94
7708	1.00	1.00	1.00	0.97	1.00	0.99	0.51	1.00	0.75	0.96	0.96	0.96
31420	1.00	1.00	1.00	1.00	1.00	1.00	0.96	0.96	0.96	0.92	0.99	0.96
42710	0.99	0.99	0.99	0.94	1.00	0.97	0.43	1.00	0.72	0.95	0.99	0.97
64100	0.97	0.99	0.98	0.95	1.00	0.97	0.97	0.90	0.94	0.96	0.99	0.98
64200	0.96	0.99	0.98	0.87	0.95	0.91	0.43	1.00	0.71	0.94	1.00	0.97
64220	1.00	1.00	1.00	0.97	0.99	0.96	0.95	0.97	0.96	0.92	1.00	0.96
64500	1.00	1.00	1.00	0.91	0.93	0.92	0.44	1.00	0.72	0.84	0.95	0.90
64350	1.00	1.00	1.00	0.94	0.96	0.95	0.80	1.00	0.90	0.90	0.94	0.92
75755	1.00	1.00	1.00	0.94	0.98	0.96	0.76	1.00	0.88	0.94	0.97	0.96
mean	0.98	0.99	0.99	0.94	0.98	0.96	0.69	0.98	0.84	0.93	0.97	0.95

**Table 3:** Sensitivity, specificity, and AUC values for the prediction models using an MDDR2 dataset.

Activity Index	CNNfp			NaïveB			RBFN			LSVM		
	Sens	Spec	AUC	Sens	Spec	AUC	Sens	Spec	AUC	Sens	Spec	AUC
9249	1.00	1.00	1.00	0.91	0.99	0.95	0.82	0.98	0.90	0.95	0.97	0.96
12455	1.00	1.00	1.00	0.88	0.97	0.92	0.66	0.98	0.82	0.93	0.96	0.94
12464	1.00	1.00	1.00	0.85	0.99	0.92	0.75	0.95	0.85	0.89	0.97	0.93
31281	1.00	1.00	1.00	0.94	1.00	0.97	0.53	1.00	0.76	0.95	0.97	0.96
43210	0.99	0.99	0.99	0.84	0.99	0.91	0.78	0.97	0.87	0.93	0.96	0.94
71522	1.00	1.00	1.00	0.82	0.99	0.91	0.75	0.97	0.86	0.91	0.97	0.94
75721	1.00	1.00	1.00	0.91	0.99	0.95	0.86	0.98	0.92	0.96	0.97	0.96
78331	0.98	0.99	0.99	0.81	0.96	0.89	0.79	0.93	0.86	0.81	0.96	0.88
78348	0.99	0.99	0.99	0.65	0.99	0.82	0.74	0.96	0.85	0.88	0.97	0.92
78351	0.99	0.99	0.99	0.82	0.94	0.88	0.59	0.96	0.78	0.91	0.95	0.93
mean	0.99	0.99	0.99	0.84	0.98	0.91	0.73	0.97	0.85	0.91	0.97	0.94

**Table 4:** Sensitivity, specificity, and AUC values for the prediction models using a Sutherland dataset.

Activity Class	CNNfp			NaïveB			RBFN			LSVM		
	Sens	Spec	AUC	Sens	Spec	AUC	Sens	Spec	AUC	Sens	Spec	AUC
Estrogen receptor	1.00	1.00	1.00	1.00	1.00	1.00	0.62	0.70	0.64	0.98	1.00	0.99
Dihydrofolate reductase	0.99	0.99	0.99	0.99	1.00	0.99	0.86	0.80	0.84	0.90	0.98	0.94
Cyclooxygenase-2 inhibitors	1.00	1.00	1.00	1.00	0.99	1.00	0.93	0.76	0.84	1.00	0.99	0.99
Benzodiazepine receptor	1.00	1.00	1.00	0.94	0.61	0.78	0.99	0.65	0.82	0.95	0.92	0.93
mean	0.99	0.99	0.99	0.98	0.90	0.94	0.85	0.73	0.79	0.95	0.97	0.96

**Table 5:** MDDR activity classes for DS1 dataset.

Activity Index	Activity Class	Active Molecules	Pairwise Similarity
07707	Adenosine agonists A1	207	0.229
07708	Adenosine agonists A2	156	0.305
31420	Rennin inhibitors	1130	0.290
42710	CCK agonists	111	0.361
64100	Monocyclic_-lactams	1346	0.336
64200	Cephalosporins	113	0.322
64220	Carbacephems	1051	0.269
64500	Carbapenems	126	0.260
64350	Tribactams	388	0.305
75755	Vitamin D analogues	455	0.386

**Table 6:** MDDR activity classes for DS2 dataset.

Activity Index	Activity Class	Active Molecules	Pairwise Similarity
09249	Muscarinic (M1) agonists	900	0.111
12455	NMDAreceptor antagonists	1400	0.098
12464	Nitric oxide synthase inhibitor	505	0.102
31281	Dopamine hydroxylase inhibitors	106	0.125
43210	Aldose reductase inhibitors	957	0.119
71522	Reverse transcriptase inhibitors	700	0.103
75721	Aromatase inhibitors	636	0.110
78331	Cyclooxygenase inhibitors	636	0.108
78348	Phospholipase A2 inhibitors	617	0.123
78351	Lipoxygenase inhibitors	2111	0.113

**Table 7:** Sutherland activity classes.

Activity Class	Active Molecules	Pairwise Similarity
Estrogen receptor	141	0.468
Ddihydrofolate reductase	393	0.502
Cyclooxygenase-2 inhibitors	303	0.687
Benzodiazepine receptor	306	0.536

**Table 8:** Details of every matrix size for every fingerprint.

Fingerprint	Features Size	$\sqrt{Features\;Size}$	Mol2mat Size *n* × *n*
ALOGP	120	10.95	11 × 11
CDK	1024	32	32 × 32
ECFC4	1024	32	32 × 32
ECFP4	1024	32	32 × 32
EPFP4	1024	32	32 × 32
GOFP	1024	32	32 × 32
PCFP	881	29.68	30 × 30
MDL	166	12.88	13 × 13

**Table 9:** Details of the first and second fully connected layers for every combination.

Combined Case	Combined Layer Size	Number of Nodes in 1st Fully Connected Layer	Number of Nodes in 2nd Fully Connected Layer
2 Fingerprints	6272	128	64
3 Fingerprints	9408	256	128
4 Fingerprints	12,544	512	256
5 Fingerprints	15,680	1024	512

## Data Availability

The MDL Drug Data Report (MDDR) dataset is owned by www.accelrys.com, accessed on 15 January 2020. A license is required to access the data.
